# Sex Bias in Susceptibility to MCMV Infection: Implication of TLR9

**DOI:** 10.1371/journal.pone.0045171

**Published:** 2012-09-20

**Authors:** Stephanie Traub, Olivier Demaria, Lionel Chasson, Fabienne Serra, Benoit Desnues, Lena Alexopoulou

**Affiliations:** 1 Centre d'Immunologie de Marseille-Luminy (CIML), Aix-Marseille Université UM 2, Marseille, France; 2 Institut National de la Santé et de la Recherche Médicale (INSERM) UMR 1104, Marseille, France; 3 Centre National de la Recherche Scientifique (CNRS), UMR 7280, Marseille, France; National Institute of Allergy and Infectious Diseases - Rocky Mountain Laboratories, United States of America

## Abstract

Toll-like receptor (TLR)-dependent pathways control the activation of various immune cells and the production of cytokines and chemokines that are important in innate immune control of viruses, including mouse cytomegalovirus (MCMV). Here we report that upon MCMV infection wild-type and TLR7^−/−^ male mice were more resistant than their female counterparts, while TLR9^−/−^ male and female mice showed similar susceptibility. Interestingly, 36 h upon MCMV infection TLR9 mRNA expression was higher in male than in female mouse spleens. MCMV infection led to stronger reduction of marginal zone (MZ) B cells, and higher infiltration of plasmacytoid dendritic cells and neutrophils in wild-type male than female mice, while no such sex differences were observed in TLR9^−/−^ mice. In accordance, the serum levels of KC and MIP-2, major neutrophil chemoattractants, were higher in wild-type, but not in TLR9^−/−^, male versus female mice. Wild-type MCMV-infected female mice showed more severe liver inflammation, necrosis and steatosis compared to infected male mice. Our data demonstrate sex differences in susceptibility to MCMV infection, accompanied by a lower activation of the innate immune system in female mice, and can be attributed, at least in a certain degree, to the lower expression of TLR9 in female than male mice.

## Introduction

Fundamental differences associated with the sex of an individual exist at every biological level, including the immune system. In general, females respond to infection, vaccination and trauma with increased antibody production, and suffer a higher incidence of autoimmune diseases, whereas inflammation is usually more severe in males, resulting in an increased mortality upon infection [1], [2], [3]. Males are hypothesized to be more susceptible to infection than females not only because androgens can modulate immunocompetence, but because sex steroid hormones affect disease resistance genes and behaviors [2]. Nevertheless, the final outcome of an infection, susceptibility or resistance, does not depend only on the sex of the infected organism, but also on the infectious agent. For example, among those individuals that have not received antiretroviral therapy, HIV infection is associated with significantly shorter survival in women than in men [4]. Studies with rodents have also revealed sex differences in susceptibility to infection for many viruses, including herpes simplex virus, vesicular stomatitis virus, coxsackievirus B3, and Theiler's murine encephalomyelitis virus [5], [6], [7], [8].

Human CMV, a member of the herpes virus family prevalent in most human populations, is rarely symptomatic in immune-competent individuals, but can cause life-threatening disease in immunodeficient hosts, as well as brain damage and hearing loss in congenitally infected children [9]. Since there is species tropism, murine cytomegalovirus (MCMV) has become a animal model for systemic human CMV infection, and has contributed greatly to our understanding of the molecular determinants of pathogenesis. MCMV is commonly administered in experimental animals via i.p. injection and can infect many different organs and cell types. In acute lethal MCMV infection the spleen and the liver are the principal sites of early viral replication, and lethality is often associated with destruction of the liver [10]. MCMV infection in the spleen follows a very distinctive pattern, beginning in the marginal zone (MZ) and spreading into the red pulp by 17 h, while by 48 h after infection there is a widespread infection both in the spleen and liver with generation of infected cells [11]. Control of MCMV infection requires both innate and adaptive host immune responses, with type I IFN (IFN-α/β) signaling serving as a key component of innate immunity. Although many cell types can produce type I IFNs in response to viral infection, plasmacytoid dendritic cells (pDCs) are considered the major producers of IFN-α in response to infection with various viruses, including MCMV [12]. Indeed, splenic pDCs are responsible for the high IFN-α/β production that is observed at 36 h upon MCMV infection in the sera of C57BL/6 mice [13]. IFN-α and NK cells largely limit early viral replication in the mouse spleen, whereas T cells are required for eventual control of acute infection and reactivation from latency. In addition to type I IFNs, other cytokines play also an important role on the mounting of an efficient host response against MCMV, including IL-12, TNF, lymphotoxin α/β, IL-6 and IFN-γ [14].

Although initially neutrophils were considered as the first line of defense mainly against bacterial infections by ingesting and killing invading microorganisms, further studies have uncovered a more general and important role of neutrophils in shaping the immune responses and contributing in the repair of tissue, as well as its breakdown, upon bacterial or viral infections [15]. The implication of neutrophils in CMV infection has also been explored in some studies. For example, MCMV-infected neutrophils display reduced chemotactic and phagocytic activity, while infection of human neutrophils with CMV by contact with CMV-infected pulmonary artery endothelial cells, increases neutrophil effector functions [16], [17]. CMV-infected endothelial cells can recruit neutrophils by the secretion of IL-8 and GROα chemokines, and transmit the virus to them by direct cell-to-cell contact and during neutrophil transendothelial migration [18]. Rodents lack a direct homologue of IL-8, but the chemokines KC and MIP-2 are regarded as functional homologues of IL-8 and are the most critical factors for the recruitment of neutrophils at the site of infection or inflammation [19].

The study of wild-type (WT) and genetically modified mice upon MCMV infection has been particularly useful in elucidating the role of antiviral innate and adaptive immune response mechanisms, and in combination with the discovery of TLRs have advanced our comprehension of host defense against MCMV infection [12], [20]. TLRs are a family of conserved transmembrane molecules that detect microbial components and play a pivotal role in shaping both innate and adaptive responses. From the 13 mammalian TLRs (10 in humans and 12 in mice), the ones that are located in the plasma membrane recognize mainly bacterial components, while the TLRs that are found within endosomal-lysosomal compartments are specialized on the detection of nucleic acid-based ligands. The endosomal TLRs include, TLR3 that detects viral double-stranded RNA, TLR7 and TLR8 that sense viral single-stranded RNA and TLR9 that responds to unmethylated CpG-containing DNA, which is found in the genomes of virus and bacteria [21], [22], [23], [24]. TLR7, TLR8 and TLR9 signal through the adaptor molecule MyD88 that leads to the production of inflammatory cytokines, while TLR3 signals through Toll/IL-1R domain-containing adapter inducing IFN-β (TRIF) [20]. The TLR9-MyD88 signaling pathway is critical for rapid MCMV clearance, since mice deficient for TLR9 (TLR9^−/−^) or MyD88 (MyD88^−/−^) are highly susceptible to MCMV infection [25], [26]. TLR3^−/−^ and TRIF^−/−^ mice show increased MCMV replication, however, the TLR3-TRIF pathway appears less important than TLR9 [26], [27]. Upon MCMV infection, although TLR7^−/−^ mice show normal survival rates, double TLR7/TLR9^−/−^ mice show higher susceptibility and viral titers than TLR9^−/−^ mice, suggesting that TLR7 also participates in antiviral host defense to MCMV [28].

In the present study, we investigated the influence of sex on acute MCMV infection in laboratory mice. We found that C57BL/6 WT male mice show higher resistance to MCMV infection compared to female counterparts and this sex difference is absent in TLR9^−/−^, but not in TLR7^−/−^ mice. MCMV infection in male and female mice revealed higher expression of TLR9 in male spleens that was accompanied with stronger reduction of the MZ B cell compartment and higher infiltration of pDCs and neutrophils. The reduction of MZ B cells was TLR9-independent, while the mobilization of neutrophils in the spleen was TLR9-dependent. These results reveal sex differences in MCMV infection, with higher susceptibility and lower activation of the immune system in female mice, which in part can be attributed to the lower expression of TLR9 in female than male mice.

## Results

### Sex differences in survival upon MCMV infection

**Figure 1:**
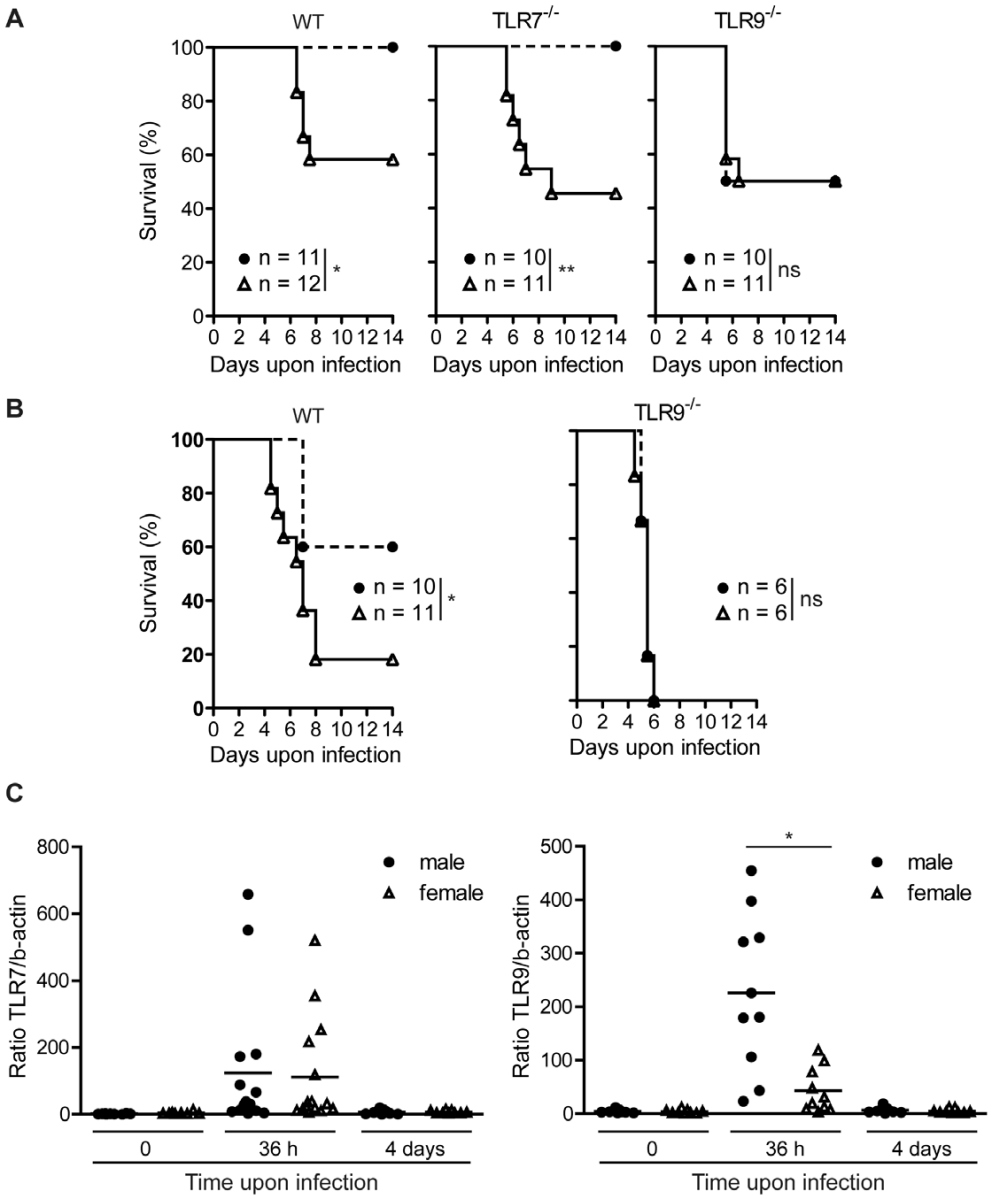
Increased survival and splenic TLR9 expression of male mice upon MCMV infection. WT, TLR7^−/−^ and TLR9^−/−^ male (black circles) and female (white triangles) mice were infected i.p with (A) 1×10^5^ PFU or (B) 1.2×10^5^ PFU of MCMV and monitored twice daily for mortality. WT and TLR7^−/−^ male mice showed statistically increased survival compared to female counterparts, while no such sex difference was observed with TLR9^−/−^ mice. (C) WT male and female mice were infected i.p. with 1×10^5^ PFU of MCMV or left untreated. Spleens were harvested 36 h or 4 days later, total RNA was extracted and the expression of TLR7, TLR9 and β-actin mRNA was determined by Q-PCR. Splenic expression of TLR7 and TLR9 is increased 36 h upon MCMV infection, and TLR9 is expressed statistically significant higher in male than female mice. Data in (A) are representative of three independent experiments; in (B) and (C) two independent experiments have been pooled together. In (C) each point represents an individual mouse. *p<0.05; **p<0.0; ns, not significant; and n, number of mice per group.

To study sex differences in susceptibility to MCMV infection, age-matched WT, TLR7^−/−^ and TLR9^−/−^ male and female mice, all in the C57BL/6 background, were infected i.p. with MCMV. Upon infection with 1×10^5^ PFU of MCMV, all WT and TLR7^−/−^ male mice survived, while female WT and TLR7^−/−^ mice were susceptible with survival rates, 58% and 45%, respectively (Fig. 1A). However, both TLR9^−/−^ male and female mice showed the same susceptibility to MCMV infection with a survival rate of 50% (Fig. 1A). Next, WT and TLR9^−/−^ mice were infected with a higher dose, 1.2×10^5^ PFU of MCMV. Upon this dose, WT male mice showed intermediate susceptibility (60% survival), WT female mice had significant reduced survival (18%), while male and female TLR9^−/−^ mice showed similar susceptibility and died by day 6 (Fig. 1B). Our data confirmed previous studies regarding the importance of TLR9 in innate immune defense against MCMV infection [26], and that TLR7^−/−^ MCMV-infected mice show similar survival rates like control mice [28]. Moreover, we found that upon MCMV infection, WT and TLR7^−/−^ male mice are more resistant than female counterparts, while TLR9^−/−^ male and female mice show similar survival rates. Thus, there is a sex difference regarding susceptibility to MCMV infection, and this difference seems to be TLR9-, but not TLR7-dependent.

### Differences in TLR9 expression in MCMV-infected male and female mice

Next, we tested whether the differences in survival upon MCMV infection between WT male and female mice, could be explained by a differential expression of TLRs. To do so, WT male and female mice were left untreated or infected with MCMV and the expression of TLR7 and TLR9 in mouse spleens was determined by quantitative PCR (Q-PCR) at 36 h and 4 days upon the infection. Spleens derived from uninfected mice showed low and similar levels of TLR7 and TLR9 mRNA expression between the two sexes (Fig. 1C and D). Interestingly, 36 h upon MCMV infection there was a dramatic increase in splenic TLR7 and TLR9 mRNA expression, whereas male mice showed an average of 5 times higher TLR9 expression than female mice, while the expression of TLR7 was similar in both sexes (Fig. 1C). Four days after MCMV infection the levels of TLR7 and TLR9 expression were back to basal levels, both in male and female spleens (Fig. 1C). Thus, MCMV infection leads to a significant up-regulation of TLR7 and TLR9 mRNA expression in mouse spleens 36 h upon MCMV infection, and TLR9 expression is significantly higher in male than female mice.

### Similar viral organ load and cytokine production in MCMV-infected male and female mice

**Figure 2:**
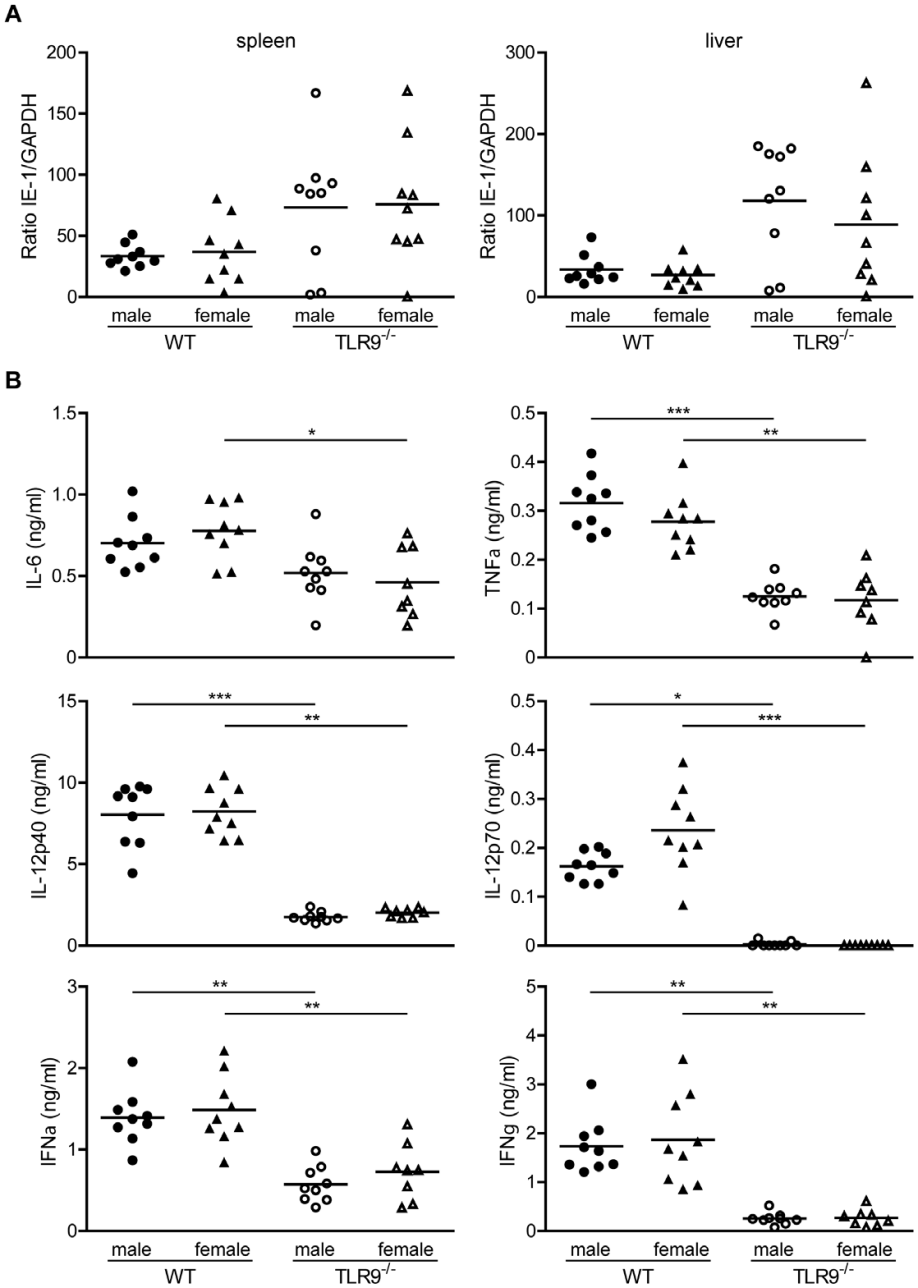
Similar viral load and cytokine production in male and female mice. WT and TLR9^−/−^ male and female mice (n = 8–9) were infected i.p. with 1×10^5^ PFU of MCMV. (A) Four days after infection the viral load in spleen and liver was determined by Q-PCR with specific primers for IE-1 and GAPDH. For each genotype there is similar viral load between male and female mice, and TLR9^−/−^ mice showed increased viral load compared to WT counterparts. (B) Sera were collected 36 h after infection and the production of IL-6, TNFα, IL-12p70 and IFNγ were measured by CBA flex, and IFNα and IL-12p40 by ELISA. For each genotype there is similar cytokine levels between male and female mice, but statistically decreased cytokine levels in TLR9^−/−^ sera compared to WT mice. Data are representative of three independent experiments. *p<0.05, **p<0.01 and ***p<0.001.

To determine whether the difference in MCMV susceptibility between male and female mice is due to difference in viral replication, we determined by Q-PCR the viral load in spleen and liver of WT and TLR9^−/−^ mice four days after MCMV infection. WT or TLR9^−/−^ male mice showed no significant difference in organ viral load compared with their female counterparts (Fig. 2A). However, and in accordance with previously published studies [26], [28], TLR9^−/−^ male and female mice showed increased viral load compared to their WT controls (Fig. 2A). Based on these results we wondered whether there was a difference in viral load at earlier time points upon MCMV infection. So, we tested the viral load in spleen and liver of WT mice on day 2 and 3 upon MCMV infection by plaque assay on confluent monolayers of 3T3 cells, and again female and male mice showed similar viral load (data not shown).

Next, we assessed the protein levels of IL-6, TNF, IL-12p40, IL-12p70, IFN-α and IFN-γ in mouse sera derived from WT or TLR9^−/−^ male and female mice, that have been infected with MCMV for 36 h. No differences were observed between WT or TLR9^−/−^ male and female mice, however, TLR9^−/−^ mice produced much lower levels of all cytokines measured compared to WT countreparts (Fig. 2B). Thus, the difference in susceptibility between WT male and female mice upon MCMV infection cannot be attributed to altered viral replication or cytokine production between the two sexes.

### Differences in the proportion of pDCs and MZ B cells in the spleens from MCMV-infected WT male and female mice

**Figure 3:**
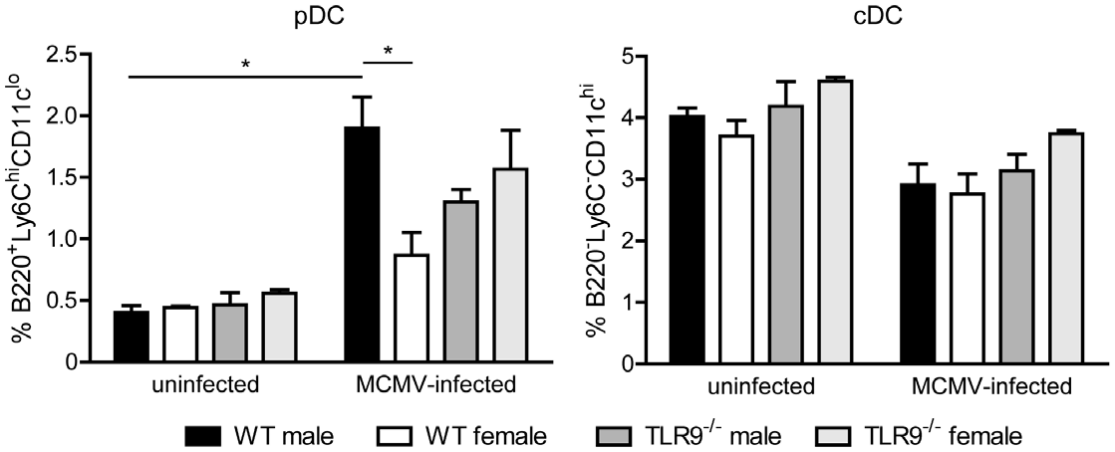
Increased proportion of splenic pDCs in MCMV-infected WT male mice. WT and TLR9^−/−^ male and female mice were left uninfected or infected i.p. with 1×10^5^ PFU of MCMV and spleens were harvested 36 h later. Erythrocyte-depleted splenocytes were analyzed by flow cytometry for the expression of B220, CD11c and Ly6C. Upon MCMV-infection the percentage of pDCs (B220^+^Ly6C^hi^CD11c^lo^) is increased in all four mouse groups, while the percentage of cDCs (B220^−^Ly6C^−^CD11c^hi^) is slightly decreased. Interestingly, upon MCMV infection WT male mice showed statistically significant increased percentage of pDCs compared to WT females. Percentages on pDCs and cDCs are presented as mean ± SEM *p<0.05. The data are representatives of 3 mice per group and of 3 independent experiments.

Both pDCs and conventional DCs (cDCs) play an important role in antiviral immunity, including MCMV infection [13], [29], so we examined these two cells types. The percentage of the pDC population (B220^+^Ly6C^high^CD11c^low^) and cDCs (B220^−^Ly6C^−^CD11c^hi^) in uninfected WT and TLR9^−/−^ male and female mice were similar (Fig. 3). Upon MCMV infection the percentage of cDCs was slightly decreased, but in a similar degree in all four mouse groups. In contrast, the percentage of pDCs was increased in MCMV-infected WT male and TLR9^−/−^ male and female mice, while WT female mice showed much lower increase compared to uninfected mice (Fig. 3). Moreover, in WT MCMV-infected mice the percentage of pDCs was statistically much higher in male than in female mice, while no such difference was observed between TLR9^−/−^ male and female mice (Fig. 3).

The micro architecture of secondary lymphoid organs, like the spleen, facilitates effective communication between antigen-presenting cells and T lymphocytes to mount protective immunity to pathogens. To examine if the differences in survival between male and female mice upon MCMV infection were correlated to differences in the number of certain cell populations, we assessed the percentage of various cell types in the spleens of WT or TLR9^−/−^ uninfected or MCMV infected mice. No obvious differences were found regarding the numbers of B, T (CD4 and CD8) and NKT cells, between male and female mice before or after infection (Table 1). Regarding, NK cells we observed no sex differences in uninfected mice, while upon MCMV infection the number of NK splenic cells was reduced but in the same degree in WT and TLR9^−/−^ male and female mice (Table 1).

**Table 1:** Frequency of splenic B, T, NK, and NKT cell subsets in untreated or MCMV-infected WT or TLR9^−/−^ male and female mice.

	Uninfected				MCMV-infected			
Cell type	WT		TLR9^−/−^		WT		TLR9^−/−^	
	male	female	male	female	male	female	male	female
B cells	63.1±0.9	60.8±1.6	58.2±3.5	61.8±1.7	63.5±1.4	61.4±4.0	64.6±1.2	63.2±1.2
CD4 T cells	51.3±2.9	49.8±3.7	46.0±7.4	48.3±2.1	51.5±3.6	47.3±1.4	50.3±4.9	44.5±1.4
CD8 T cells	17.0±0.4	17.9±1.3	16.9±1.3	20.7±2.2	18.3±0.6	19.5±3.3	16.4±1.1	21.8±1.8
NK cells	1.9±0.5	2.4±0.5	2.2±0.7	2.0±0.2	1.4±0.2	1.6±0.5	1.5±0.3	2.0±±0.4
NKT cells	1.3±0.1	1.4±0.1	1.1±0.2	1.2±0.1	1.2±0.2	1.0±0.1	1.2±0.2	1.2±0.2

WT and TLR9^−/−^ male and female mice were left uninfected or infected i.p. with 1,2×10^5^ PFU of MCMV. After, 36 h spleens were harvested, total splenocytes were isolated, stained for B220, CD19, CD3, CD4, CD8, or NK1.1 and analyzed by flow cytometry. The frequency of B cells (CD19^+^B220^+^), CD4 (CD3^+^CD4^+^) and CD8 (CD3^+^CD8^+^) T cells, and NK (B220^−^NK1.1^+^CD3^−^), and NKT (B220^−^NK1.1^+^CD3^−^) cells was determined after gating among live cells. All values denote percentages (mean ± SD) of three mice per group, and are representative of two or three independent experiments.

**Figure 4:**
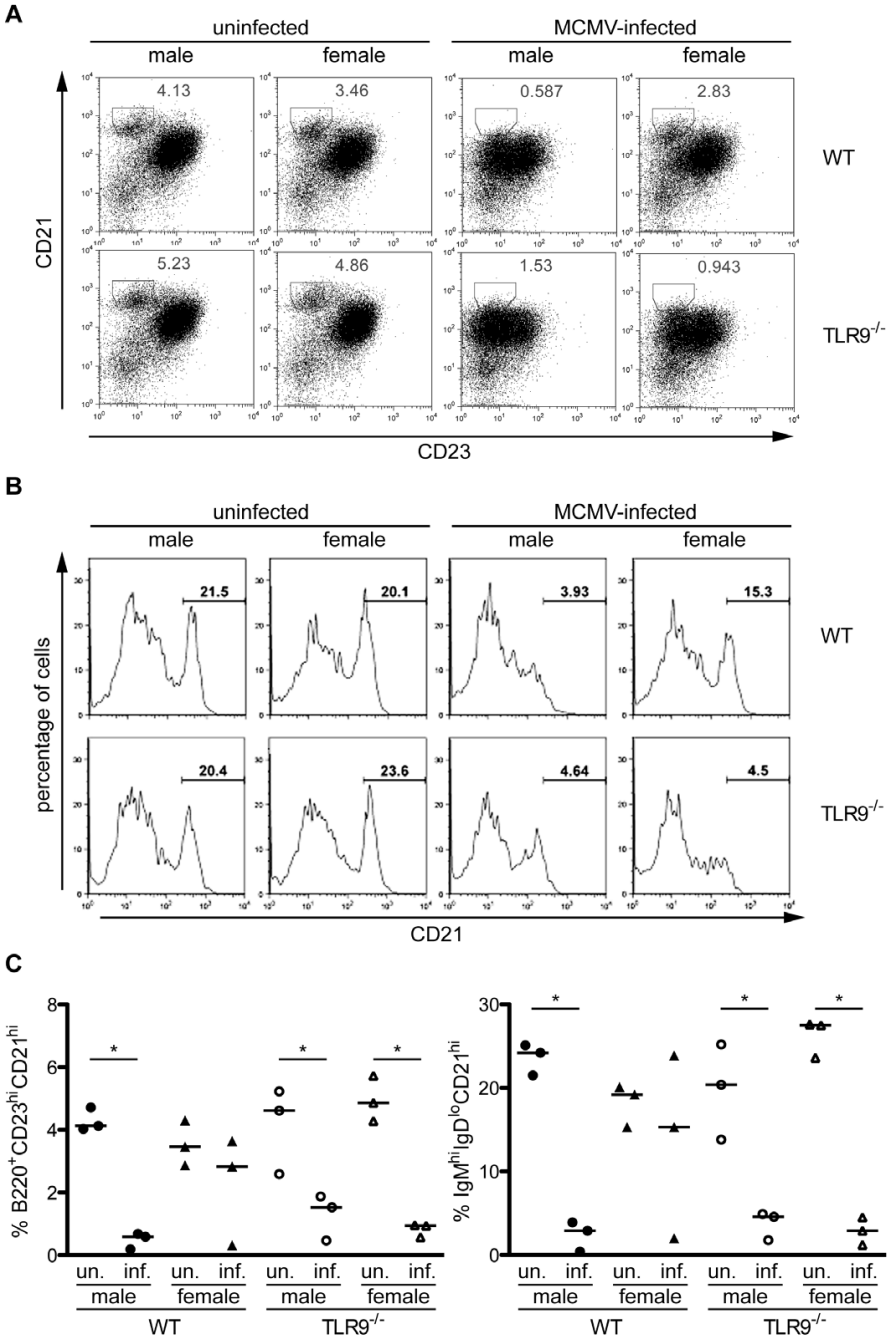
Differences in the proportion of MZ B cells in MCMV-infected WT male and female mice. WT and TLR9^−/−^ male and female mice were left uninfected or infected i.p. with 1×10^5^ PFU of MCMV and spleens were harvested 36 hours later. Erythrocyte-depleted splenocytes were analyzed by flow cytometry for the expression of B220, CD19, CD23, CD21, IgM and IgD. The MZ B cell population, B220^+^CD19^+^CD23^lo^CD21^hi^ on (A) or IgM^high^IgD^low^CD21^high^ on (B), of MCMV-infected WT male and TLR9^−/−^ male and female mice was dramatically reduced compared to uninfected counterparts, while this reduction was minor in MCMV-infected female mice. (A) Plots show percentage of MZ B cells (CD23^lo^CD21^hi^) on B220^+^ gated lymphocytes. (B) Histograms show expression of CD21 on IgM^high^IgD^low^ B cells. (C) Graph with the numeric data of all 3 mice per group that were used for the experiments in (A) and (B). *p<0.05. The data are representatives of 3 independent experiments.

Since MZ B cells constitute the first line of defense against blood-borne pathogens in the spleen and MCMV infection in the spleen begins in the MZ [11], we next evaluated the MZ B cell compartment. We found that 36 h after MCMV infection the MZ B cell population (B220^+^ CD19^+^CD23^low^CD21^high^) in WT male and TLR9^−/−^ male and female mice was significantly reduced compared to uninfected counterparts, while this reduction was minor in MCMV-infected WT female mice (Fig. 4A and C). This phenotypic defect was further confirmed by the reduction of CD21^high^ cells among the IgM^high^IgD^low^ B lymphocytes, a characteristic trait of MZ B cells (Fig. 4B and C). Thus, upon MCMV infection WT female mice show reduced recruitment of splenic pDCs and mobilization of the MZ B cells compared to WT male mice, but these sex differences are absent in TLR9^−/−^ mice.

### Differences in neutrophil attraction in MCMV-infected WT male and female mice

**Figure 5:**
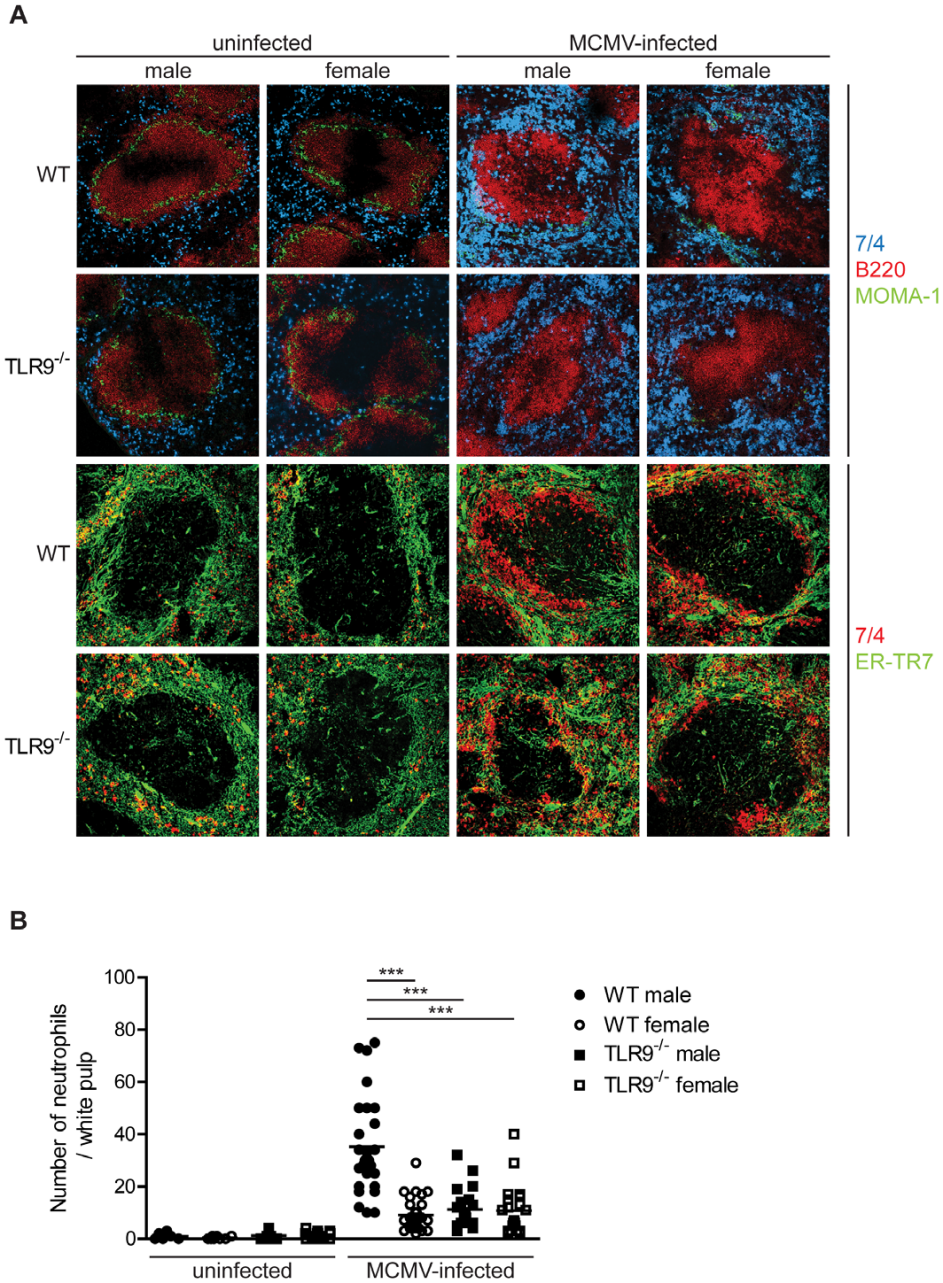
Increased number of neutrophils in the splenic white pulp of MCMV-infected WT male mice. WT or TLR9^−/−^ male and female mice were left untreated of infected with 1×10^5^ PFU of MCMV and 4 days later spleens were collected and processed for immunofluorescent analysis. (A) Murine spleen sections were incubated with antibodies specific to neutrophils (7/4, blue or red), B cells (B220, red), marginal metalophilic macrophages (MOMA-1, green) and reticular fibroblasts (ER-TR7, green). MCMV infection led to (A) a dramatic increase in the number of neutrophils that were present in the splenic red pulp and disappearance of the MZ metallophilic macrophages and (B) an increase in the number of neutrophils in the splenic white pulp areas compared to uninfected mice. These phenomena were more prominent in WT male than in WT female or TLR9^−/−^ male and female mice. (B) Number of neutrophils per white pulp area were counted on slides stained in A. *** p<0.001. Data are representative of 9 mice per group.

Neutrophils are the first major population of leukocytes to infiltrate infected or injured tissues and are crucial for initiating host innate defense and adaptive immunity [15]. Interestingly, several studies have identified altered function of neutrophils during animal CMV infection [30], [31]. In order to evaluate the splenic distribution of neutrophils upon MCMV infection, the spleens from WT and TLR9^−/−^ male and female mice that have been infected with MCMV or left untreated were analyzed by immunofluorescence. We found that 4 days after MCMV infection there was a dramatic increase in the number of neutrophils that were present in the splenic red pulp compared to uninfected mice (Fig. 5A). Moreover, MCMV infection led to an almost complete disappearance of the MZ metallophilic macrophages, in accordance with previous published data [32], and an increase in the number of neutrophils in the white pulp areas compared to uninfected mice (Fig. 5A). Indeed, the number of infiltrating neutrophils in the white pulp areas of infected WT male mice were significant higher compared to WT female or TLR9^−/−^ male and female mice (Fig. 5B). Furthermore, splenic sections were stained with 7/4 and ER-TR7 antibodies. ER-TR7 stains fibroblasts, connective tissue and endothelial cells of blood vessels and helps to discriminate the white and red pulp area of the spleens. Analysis of this staining showed that MCMV-infection induced a compact ring shape accumulation of neutrophils at the periphery of the white pulp areas in WT male mice, while this accumulation was dramatically reduced in WT female and TLR9^−/−^ male and female mice (Fig. 5A).

**Figure 6:**
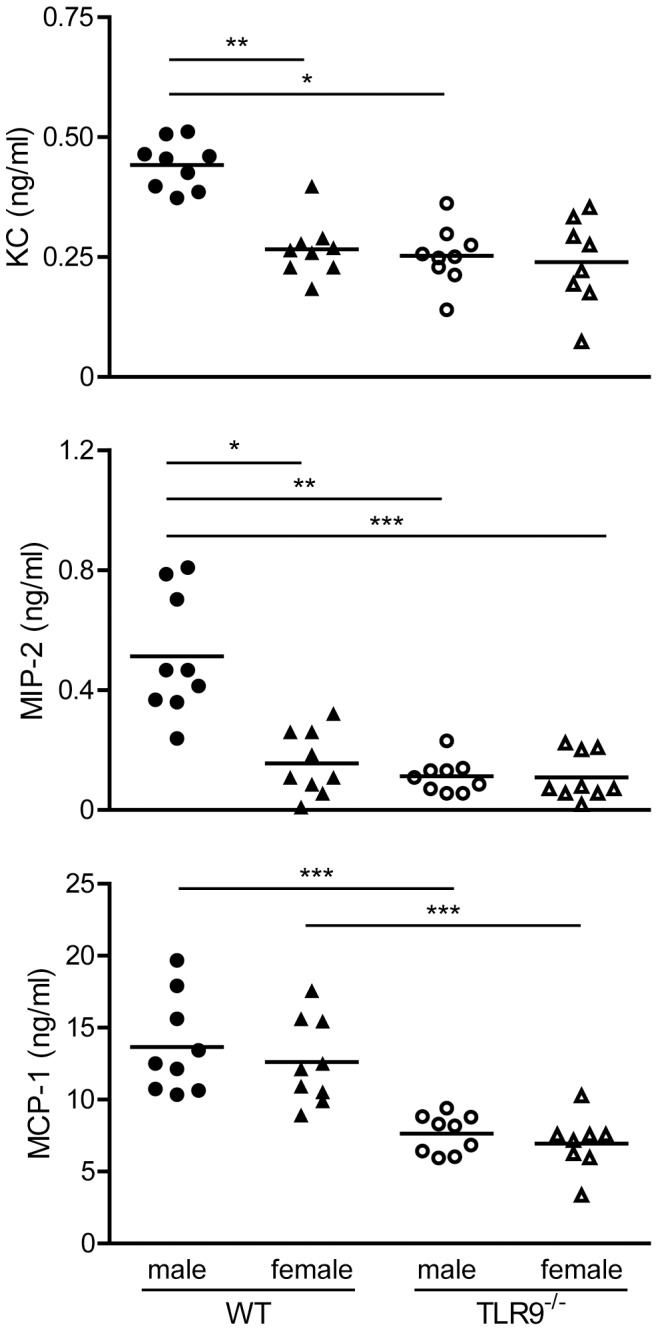
Increased levels of the neutrophil attractant chemokines KC and MIP-2 in WT male mouse sera upon MCMV infection. WT or TLR9^−/−^ male and female mice were infected i.p. with 1×10^5^ PFU MCMV and sera were collected 36 h after infection. Upon MCMV infection, WT male mice produced statistically significant higher levels of KC and MIP-2 than WT female or TLR9^−/−^ male and female mice, while the MCP-1 levels were higher in WT mice compared to TLR9^−/−^ mice. KC, MIP-2 and MCP-1 protein levels were measured by CBA flex or ELISA. Data are representative of four independent experiments. *p<0.05, **p<0.01 and ***p<0.001.

Neutrophils are attracted to the infected tissue by chemokines such as KC or MIP-2, while monocytes are attracted by MCP-1, so we assessed the protein levels of KC, MIP-2 and MCP-1 in the sera of uninfected or MCMV-infected WT or TLR9^−/−^ male and female mice at 36 h upon infection. We observed significant higher levels of KC and MIP-2 in WT male than in WT female and TLR9^−/−^ male or female mice (Fig. 6). However, no differences were observed regarding the release of MCP-1 between WT male and female mice, although the levels of MCP-1 in TLR9^−/−^ mice were decreased compared to WT controls (Fig. 6). Thus, we observed a decreased attraction of neutrophils in the spleens of MCMV-infected WT female and TLR9^−/−^ mice compared to WT male mice, which was associated with lower serum levels of KC and MIP-2, the major chemo-attractants of neutrophils.

### MCMV-induced liver pathology is more severe in WT female than male mice

**Figure 7:**
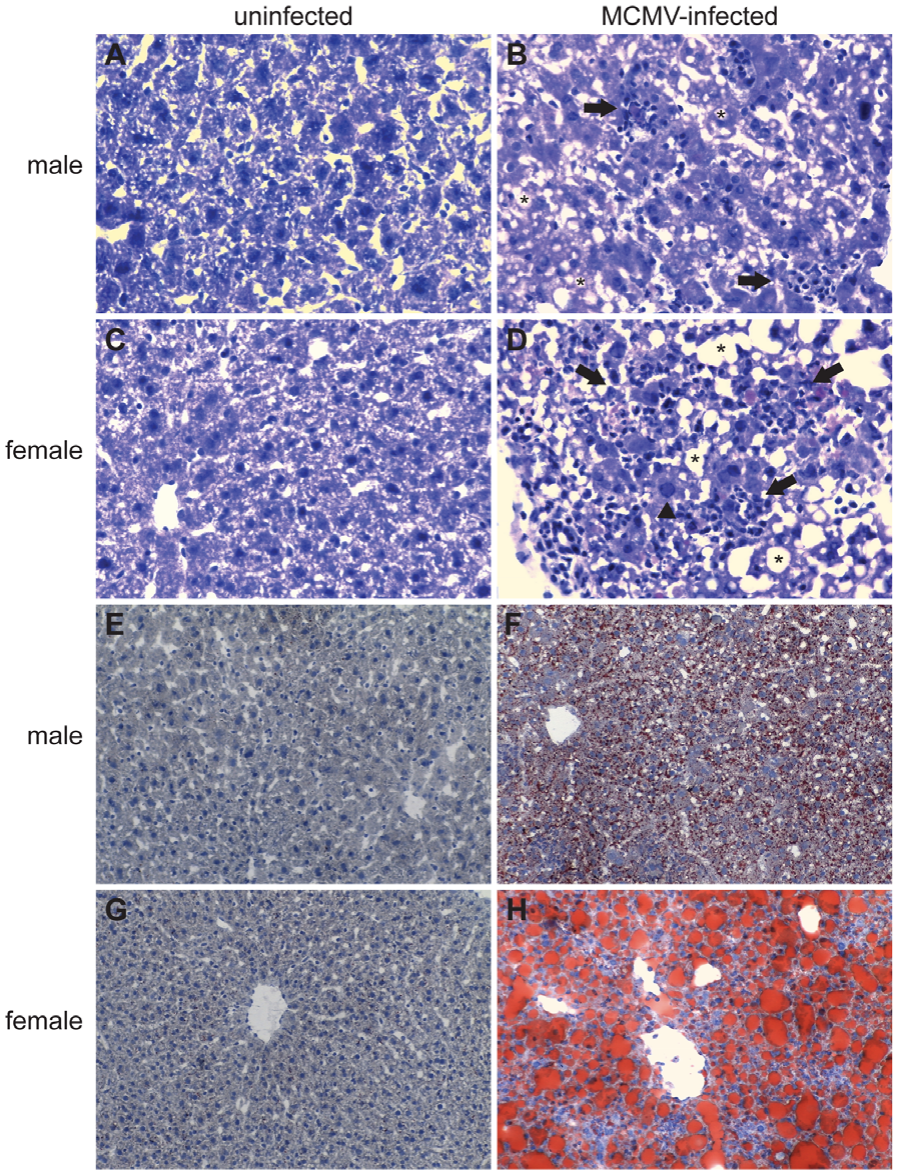
Increased liver inflammation and steatosis in MCMV-infected female mice. (A) Livers were harvested and tissue sections were prepared from uninfected (A, C, E and G) or day 3 MCMV-infected male (B and F) and female (D and H) mice as described in Materials and Methods. Hematoxylin and eosin stained liver sections from uninfected (A and C) or MCMV infected (B and D) mice revealed inflammatory and necrotic foci in MCMV-infected mice. Female infected mice had increased inflammation and necrosis (more numerous and bigger foci) compared to male mice. Arrows identify inflammatory and necrotic foci, arrowhead shows an hepatocyte with an enlarged nucleous (karyomegaly) which contains large eosinophilic intranuclear inclusion and pheripheralized chromatin, asterisks indicate intracytoplasmic clear vacuoles. Accumulation of lipids (red stained lipid droplets) was detected by Oil red O staining (E–H) and was significantly more obvious in MCMV-infected female (H) versus male (F) liver sections, while in uninfected (E and G) mice was absent. Magnifications: A–D ×40 and E–H ×20. Data are representative of 2 independent experiments and with 3 mice per group.

Previous studies have shown that liver inflammation peaks at 2–3 days after intraperitoneal infection with MCMV [33]. To determine the extent and type of liver pathology, samples were isolated from WT uninfected or day 3 MCMV-infected male and female mice. Tissue sections were prepared and stained with hematoxylin/eosin for histopathological morphological analysis. In contrast to the histological appearance of normal, uninfected liver sections (Fig. 7A and C) MCMV-infected female mice showed substantial increased liver inflammation and necrosis (Fig. 7D), compared to their male (Fig. 7B) counterparts. These data are summarized in Table 2 and representative pictures are shown in Figure 7. Moreover, round clear well demarcated cytoplasmic vacuoles characteristic of steatosis were present in MCMV-infected mice (Fig. 7B and D) and Oil red O staining confirmed that these cytoplasmic vacuoles in hepatocytes were in fact lipid deposits (Fig. 7F and H). Steatosis was more severe in female mice since the lipid vacuoles were large to very large and markedly expended the cytoplasm of hepatocytes in MCMV-infected females (Fig. 7H and Table 2), whereas in MCMV-infected males the hepatocytes were mildly to moderately expanded by smaller vacuoles (Fig. 7F and Table 2).

**Table 2:** Increased liver pathology in wild-type female compared to male mice infected with MCMV for 3 days.

Mice	Necrosis		Inflammation	Karyomegaly/Intranuclear inclusion bodies	Steatosis/lipidosis	
Mouse strain/treatment (number of mice per group)	Distribution	Severity Mean (value for each mouse)	Mean (value for each mouse)	Phenotype (number of mice/total)	Distribution	Severity Mean (value for each mouse)
WT male/control (n = 2)	Absent	-	-	-	-	-
WT male/MCMV day 3 (n = 5)	Random	2.2 (3,1,2,3,2)	2 (2,1,2,3,2)	Several (1/5) Scattered (1/5) Occasional (2/5) Absent (1/5)	Diffuse	1.2 (2,0,1,2,1)
WT female/control (n = 2)	Absent	-	-	-	-	-
WT female/MCMV day 3 (n = 5)	Random	4.6 (5,4,5,5,4)	2.8 (3,2,3,3,3)	Several (4/5) Scattered (1/5)	Diffuse	3 (3,3,3,3,3)

Wild-type male and female mice were left untreated (control) or infected with MCMV for 3 days and the following distinct measures of liver pathology were evaluated: necrotic foci, microscopic inflammatory foci and karyomegaly as revealed with H&E staining; and lipidosis as revealed by Oil red O staining. The grading system was as follows: for necrosis 1<5%, 2 = 5–10%, 3 = 10–20%, 4 = 20–40% and 5>40% of hepatocellular lobule affected; for inflammation 1 = mild, 2 = moderate, 3 = marked numbers of inflammatory cells; and lipidosis 1 = few lipid vacuoles of small size, 2 = moderate numbers of lipid vacuoles of small to medium size and 3 = numerous clear vacuoles ranging from small to very large size in hepatocytes.

## Discussion

Sex bias in human CMV infection has been observed, where in severe congenital CMV infection the risk of abnormal brain development in infected fetuses is twice as high in females than in males [34]. Moreover, in adult healthy CMV-seropositive humans the memory T helper response to CMV in significantly higher in women than men [35]. Thus, there is a sex bias to CMV infection in humans but he underlying mechanism(s) is still unknown.

The present study was undertaken to define if there is a sex difference in MCMV infection in C57BL/6 mice and to investigate the potential contribution of TLRs in this phenomenon. We found that upon MCMV infection WT and TLR7^−/−^ male mice were significantly more resistant than female mice, while TLR9^−/−^ female and male mice showed similar susceptibility (Fig. 1A and B). Based on these initial observations and the fact that TLR7 and TLR9 have been implicated, although in a different degree, in the pathophysiology of MCMV infection [26], [28], [36], we thought that differences in TLR activation between the two sexes might explain the differences in survival. We found that 36 h upon MCMV infection there was a dramatic upregulation of splenic TLR7 and TLR9 mRNA expression, where infected male mice showed 5 times higher TLR9 expression than female counterparts, while no such sex differences were observed for TLR7 (Fig. 1C). The factor(s) that lead to the differential expression of TLR9 between male and female mice upon MCMV infection remain to be elucidated. We could speculate that sex hormones might regulate the expression of TLR9, since previous studies have also identified sex differences in response to TLR stimulation that were or not attributed to sex hormones [37], [38].

To our surprise, the difference in survival between WT male and female mice upon MCMV infection could not be attributed to differences in viral load or cytokine production (Fig. 2). However, the decreased survival of MCMV-infected WT female versus male mice could be attributed to increased overall liver damage, including inflammation, necrosis and accumulation of lipids (steatosis) (Fig. 7). The accumulation of lipids in hepatocytes is indicative of a disturbance of lipid metabolism and maybe caused by the accumulation of viral proteins [39]. Viral steatosis is directly linked to hepatitis C virus replication and disappears upon successful antiviral therapy [40]. Upon MCMV infection TLR9^−/−^ mice had increased viral load and produced statistically significant lower levels of various cytokines compared to WT mice (Fig. 2), confirming our and others previous findings that TLR9 is an important component of the innate immune defense against MCMV infection [25], [26], [28]. In accordance to our results, a recent study has also shown greater mortality in female mice upon systemic infection with large double-stranded DNA viruses. Female mice were 27-, 2.5- and 2-fold more susceptible than male mice upon infection with herpes simplex virus type I (HSV-1), MCMV or vaccinia virus, respectively [41]. Since the biggest difference was observed with HSV-1, the authors extended their studies on HSV-1 infection, and similar to our observations, they did not find any differences in serum inflammatory cytokines, or hepatic viral titers between the two sexes [41]. Although, no differences were observed in serum levels of IFNα upon HSV-1 infection, the differences observed in survival between female and male mice were nearly abrogated in the absence of IFN type I receptor signaling and substantially decreased in the absence of DAP12, an adaptor molecule that negatively regulates TLR signaling [41]. By combining the above findings we could speculate that although we did not find differences in IFNα serum levels still the difference in survival between male and female mice upon MCMV infection is due to type I IFN response that is driven by TLR9 and pDCs.

Indeed, pDCs represent an essential immune cell type for the initiation of both innate and adaptive immune responses to viral infections, and TLR9 is important for MCMV-mediated type I IFN production by pDCs [12], [13], [28]. In physiological conditions, pDCs enter organs at very low levels, however, they are far more numerous in diseased or inflamed tissues. In the current study, we found that MCMV infection induces a strong influx of pDCs in WT male spleens compared to uninfected counterparts, while in female mice this increase was less obvious (Fig. 3). Interestingly, this sex difference was not observed in TLR9^−/−^ mice. So the decreased ability of WT female versus male mice to survive MCMV infection could be correlated with the decreased rate of pDCs migration to the spleen.

MZ B cells are critical for antibody protection against bacterial and viral infections at relatively early stages of infection. MZ B cells have a partially activated phenotype, are non-recirculatory and reside in the splenic MZ [42]. Of note, diverse TLR agonists activate MZ B cells to become activated and leave the MZ through pathways that are differentially dependent on MyD88 and IFN-αβ receptor signaling [43]. Our experiments show that 36 h upon MCMV infection there is a dramatic reduction of the MZ B cell population in WT male mice, but this phenomenon is less evident in the spleens of infected female mice (Fig. 4), suggesting that MZ B cells are less activated in female mice. However, in TLR9^−/−^ mice the reduction of the MZ B cell population is similar to WT male mice, indicating that the activation of the MZ B cell compartment upon MCMV infection is not TLR9-dependent.

Neutrophils contribute to host defense against invading microorganisms are rapidly recruited to sites of infection, where they initiate inflammatory responses, enhance pathogen clearance and further the trafficking of immune cells. Although neutrophil influx into tissues is often associated with enhanced pathology, recent evidence suggest that neutrophils are also involved in the active resolution of inflammation through the production of pro-resolving lipid mediators and anti-inflammatory molecules [15], [44]. Access of the splenic white pulp, which play a major role in the development of immune responses, has been shown to be restricted to lymphocytes and DCs, and the distribution of neutrophils is almost exclusively confined to the red pulp [45]. Nevertheless, it has been reported that migration of splenic neutrophils to the T cell area of the white pulp can be induced upon injection of LPS and this entry is strictly MyD88- and CD14-dependent [46]. Similarly, we found that MCMV infection induced a substantial increase in the number of splenic neutrophils and their migration to the T cell area of the white pulp in WT male mice, and this increase was associated with elevated serum levels of the neutrophil chemoattractants MIP-2 and KC (Fig. 5 and 6). However, these phenomena were less obvious in WT female and TLR9^−/−^ mice, suggesting that the increased survival of WT male mice to MCMV infection might be in part attributed to neutrophil migration and function, a phenomenon that depends on TLR9. Of note, human neutrophils express all TLRs, except TLR3, respond to TLR agonists, including TLR9, and TLR stimulated neutrophils recruit innate immune cells to sites of infection [47]. Murine neutrophils also express TLR9 and respond to TLR9 ligand [48]. Thus, the participation of neutrophils through TLR9-activation in the clearance of MCMV can be either direct, since neutrophils can also be infected by MCMV [16], indirect through the recruitment of other immune cells or a combination of the two.

In summary, upon MCMV infection, male mice show increased survival compared to females due to decreased liver pathology and better immune responses, including increased numbers of splenic pDCs, stronger mobilization of MZ B cells and increased neutrophil migration in the splenic white pulp. Part of the protection of the male mice may be attributed to the higher expression of TLR9 and stronger mobilization of neutrophils in male versus female mice, which leads to stronger immune response against the virus. Our results might have implications that go beyond MCMV infection since differential susceptibility to several viruses, including CMV, have been described for men and women and antiviral immunity in humans have also been shown to involve TLR7, TLR8 and TLR9 [3].

## Materials and Methods

### Ethics statement

Experiments were conducted in accordance with institutional guidelines for animal care and use. Protocols were approved by the Direction Départementales des Services Vétérinaires des Bouches du Rhône.

### Mice

C57BL/6 mice were purchased from Charles River (L'Arbesle, France). TLR7^−/−^ were generated as previously described [24]. TLR9^−/−^ mice were provided by M. Dalod (CIML) upon written approval of S. Akira [23]. Both, TLR7^−/−^ and TLR9^−/−^ mice were backcrossed for 10 generations in the C57BL/6 background. Mice were housed under specific pathogen-free conditions and handled in accordance with French and European directives. INSERM guidelines have been followed regarding animal experimentation and Centre d'Immunologie de Marseille-Luminy has been credited by DDSV, the French governing body with authorization No 02875 for mouse experimentation.

### MCMV infection

MCMV strain K181 was a kind gift of Helen Farrell (Centre for Preventive Medicine, Suffolk, UK). MCMV was prepared as a homogenate of salivary glands harvested from 6 weeks old CD1 mice that have been infected with MCMV two weeks earlier. Viral titers were determined by standard plaque assay on confluent monolayers of mouse embryonic fibroblasts. Groups of age-matched mice (8 to 12 weeks old) were infected i.p. with 1 or 1.2×10^5^ PFU of MCMV per 20 g body weight.

### Quantitative PCR (Q-PCR)

Total RNA from mouse spleens was isolated with TRIzol reagent (Invitrogen, Auckland, NZ) and contaminant DNA was removed by DNase I (Ambion, Huntington, UK) according to the manufacturer's instructions. Total RNA was reversed transcribed using SuperScript II reverse transcriptase (Invitrogen). cDNA was amplified by Q-PCR using the following primers: TLR7 5′-TGGCTCCCTTCTCAGGATGA-3′ and 5′-CCGTGTCCACATCGAAAACA-3′; TLR9 5′-AGCCTGAGCCACACCAACAT-3′ and 5′-GGACGCGCAGGCTGTATAGG-3′; and β-actin 5′-CCCTGAACCCTAAGGCCAAC-3′ and 5′-GACAGCACAGCCTGGATGG-3′. For the determination of MCMV viral load, total DNA was isolated from tissues by DNeasy tissue Kit (Qiagen, Hilden, Germany). Parallel reactions were performed for the detection of DNA of immediate early gene-1 (IE-1) and GAPDH. Q-PCR was performed using Power SYBR Green PCR mastermix (Applied Biosystems) and the following primers: IE-1 5′-GGCTTCATGATCCACCCTGTT-3′ and 5′-TGCCATACTGCCAGCTGAGA-3′, GAPDH 5′-TTGCAGTGGCAAAGTGGAGA-3′ and 5′-GGCTCCCCGTTGATGACAAG-3′. Q-PCR was performed on an Applied Biosystems PRISM 7700 Sequence Detection System (Warrington, UK). The amount of target was calculated relative to the calibrator by 2^−ΔCT^, resulting in data expressing a target copy number ratio (TLR7/β-actin, TLR9/β-actin or IE-1/GAPDH).

### Measurement of cytokine and chemokine production

The protein levels of IL-6, TNF, IL-12p70, IFN-γ, KC and MCP-1 in mouse sera were measured by CBA flex (BD Bioscience, San Diego, USA), while IL-12p40 (BD OptEIA, BD Bioscience), IFNα (PBL Biomedical Laboratories, New Jersey, USA) and MIP-2 (R&D Systems, Abington, UK) were measured by ELISA according to manufacturer's instructions.

### Flow cytometry

Mouse spleens were harvested, fine minced and digested with collagenase type II (Worthington, Lakewood, NJ) and DNase I from bovine pancreas (Sigma, St. Louis, USA). Red blood cells were lysed with Red Blood lysis buffer (eBioscience, San Diego, CA). Splenocytes were stained with the indicated fluorochrome-conjugated Abs for 30 min on ice. The following Abs were used: B220, CD19, CD3, CD4, CD8, NK1.1, CD23, CD21, IgM, IgD, Ly6C and CD11c. All antibodies were from BD Bioscience (San Diego, CA, USA). After fixation cells were analyzed using a Calibur flow cytometer equipped with CellQuest (BD Bioscience). Data were analyzed by FlowJo software (Ashland, USA).

### Histology and immunofluorescence analysis

Spleens or livers were embedded in OCT-compound (Sakura Finetek, Torrance, USA) and frozen in liquid nitrogen. Sections were cut on a cryostat at 10 µm, thaw-mounted on gelatinized slides, air-dried and stored at −20°C. Immediately before use, spleen sections were fixed in acetone containing 0.03% H_2_O_2_, rehydrated in PBS and stained with the following antibodies: B220-PE (BD Bioscience), MOMA-1-FITC and 7/4-Alexa 647 (AbD Serotec) or ER-TR7 (BMA Biomedicals, Augst, Switzerland) followed by anti-rat IgG-Alexa 488 (Invitrogen). Confocal microscopy was performed with a Zeiss LSM510 microscope. Image processing was performed with Zeiss LSM software and Abobe Photoshop. Liver sections were fixed in aceton for 10 min, stained with hematoxylin and eosin (H&E) and analyzed microscopically. Necrotic foci were composed of eosinophilic amorphous material with cellular debris and shruncken hypereosinophilic hepatocytes, while inflammatory foci consisted of clusters of neutrophils and mononuclear inflammatory cells. For lipid staining, liver sections were fixed in formalin, rinsed with running water followed by 60% isopropanol, stained with 0.3% Oil red O diluted in 60% isopropanol for 15 min, rinsed with 60% isopropanol, lightly stained with haematoxylin, rinsed with running water and cover slides were mounted with a water soluble mounting media. Oil-Red-O positive deposits were visualized by light microscopy.

### Statistical analysis

Statistical analysis was performed using the GraphPad Prism program (GraphPad Software, San Diego, USA). All data are mean ± SD. Significance of differences was assessed by two-tailed unpaired t-test for two groups and by one-way ANOVA employing the Kruskal Wallis test for experiments with more than two groups. Statistical analysis for the survival curves was performed using the Log-rank/Mantel-Cox test.
